# Atherogenesis May Involve the Prooxidant and Proinflammatory Effects of Ferryl Hemoglobin

**DOI:** 10.1155/2013/676425

**Published:** 2013-05-15

**Authors:** László Potor, Emese Bányai, Gergely Becs, Miguel P. Soares, György Balla, József Balla, Viktória Jeney

**Affiliations:** ^1^MTA-DE Vascular Biology, Thrombosis and Hemostasis Research Group, Hungarian Academy of Sciences, 4012 Debrecen, Hungary; ^2^Department of Medicine, University of Debrecen, 4012 Debrecen, Hungary; ^3^Instituto Gulbenkian de Ciência, 2780-156 Oeiras, Portugal; ^4^Department of Pediatrics, University of Debrecen, 4012 Debrecen, Hungary

## Abstract

Oxidized cell-free hemoglobin (Hb), including covalently cross-linked Hb multimers, is present in advanced atherosclerotic lesions. Oxidation of Hb produces methemoglobin (Fe^3+^) and ferryl hemoglobin (Fe^4+^ = O^2−^). Ferryl iron is unstable and can return to the Fe^3+^ state by reacting with specific amino acids of the globin chains. In these reactions globin radicals are produced followed by termination reactions yielding covalently cross-linked Hb multimers. Despite the evanescent nature of the ferryl state, herein we refer to this oxidized Hb as “ferryl Hb.” Our aim in this work was to study formation and biological effects of ferrylHb. We demonstrate that ferrylHb, like metHb, can release its heme group, leading to sensitization of endothelial cells (ECs) to oxidant-mediated killing and to oxidation of low-density lipoprotein (LDL). Furthermore, we observed that both oxidized LDL and lipids derived from human atherosclerotic lesions trigger Hb oxidation and subsequent production of covalently cross-linked ferrylHb multimers. Previously we showed that ferrylHb disrupts EC monolayer integrity and induces expression of inflammatory cell adhesion molecules. Here we show that when exposed to ferrylHb, EC monolayers exhibit increased permeability and enhanced monocyte adhesion. Taken together, interactions between cell-free Hb and atheroma lipids engage in a vicious cycle, amplifying oxidation of plaque lipids and Hb. These processes trigger EC activation and cytotoxicity.

## 1. Introduction

Extracellular lipid accumulation is the main feature of type IV atherosclerotic lesions. These can progress into more complicated lesions, in which there is rupture of the fibrous cap accompanied by either hematoma/hemorrhage and thrombus formation or intraplaque hemorrhage in the neovasculature sprouting from the vasa vasorum. These events provoke the clinical symptoms and are responsible for atherosclerosis-associated morbidity and mortality [1–5].

Li et al. describe the complicated lesion as a highly oxidative environment containing products of lipid peroxidation such as lipid hydroperoxides, aldehydes, and carbonyls [6]. The authors suggest that these oxidation products are toxic for incoming cells, especially macrophages, and constitute a “death zone,” perhaps explaining the persistence and growth of atherosclerotic lesions. 

Upon plaque rupture or intraplaque hemorrhage, red blood cells (RBCs) are brought into close contact with plaque materials. Hemoglobin within RBCs is protected from oxidation because erythrocytes are equipped with highly effective antioxidant defenses [7]. Based on our previous work, lipids derived from atheromatous plaque or oxidized low-density lipoprotein (LDL) can cause RBC lysis and subsequent oxidation of Hb into metHb [8]. The effect of oxLDL and plaque lipids can be mimicked by cumene hydroperoxide. Moreover, enzymatic conversion of lipidhydroperoxides to alcohol by GSH/GPx causes significant inhibition of RBC lysis and Hb oxidation triggered by oxLDL and plaque lipids, suggesting that lipid hydroperoxides play a major role in these processes [8]. We determined the Hb composition of human complicated atherosclerotic lesions and found that as much as 50% of the total Hb content is oxidized in these lesions [8]. 

A complex interplay between Hb and peroxides exists leading to the formation of metHb (Fe^3+^), ferrylHb (Fe^4+^), and oxoferrylHb (Fe^4+^ = O^2-^) species [9, 10]. Protein radicals are transiently formed in the reactions between the ferryl or oxoferryl species and the surrounding globin chains with the involvement of specific amino acids such as *α*Tyr-24, *α*Tyr-42, *α*His-20, *β*Tyr-35, *β*Tyr-130, and *β*Cys-93 [11, 12]. Termination reactions of globin radicals can yield covalently cross-linked Hb multimers which are likely to possess unique biological properties. Previously, we described the presence of these cross-linked Hb species and elevated dityrosine levels in human complicated atherosclerotic lesions [8].

Oxidized Hb species have been suggested to act as cytotoxic pro oxidants. For example, metHb sensitizes vascular EC to oxidant-mediated killing [13]. Furthermore when metHb is present in plasma, it will promote oxidative modification of LDL [14]. The release of the prosthetic heme group is crucial in these metHb-triggered oxidative processes. In contrast, the pathophysiological effect of the highly oxidized ferrylHb is less studied, even though its presence in complicated human atherosclerotic lesions has been shown [8]. We have previously shown that ferrylHb, but not native Hb or metHb, can act as a proinflammatory product targeting vascular EC [15].

The goal of the present study was to address the potential interactions between RBC-derived Hb and atheroma lipids, focusing on the formation and biological effects of ferrylHb. Here we report that lipids derived from human atheromatous lesions and oxLDL trigger the formation of covalently cross-linked Hb multimers, which in turn activate EC. When exposed to ferrylHb, EC monolayers exhibit increased permeability and enhanced monocyte adhesion. These are the results of amplification of the oxidative reactions between Hb and atheroma lipids. Based on these observations, we propose that lysis of erythrocytes and formation of ferrylHb might contribute to the progression of atherosclerosis. 

## 2. Materials and Methods

### 2.1. Materials

Reagents were purchased from Sigma-Aldrich (St. Louis, MO) unless otherwise specified.

### 2.2. Tissue Samples

Collection of specimens of human atherosclerotic lesions from beating-heart deceased donors and extraction of lipids were performed as previously described [8]. All procedures were approved by the Scientific and Research Ethics Committee of the Scientific Council of Health of the Hungarian Government. 

### 2.3. Cell Culture

Human umbilical vein EC (HUVECs) were removed by exposure to dispase and cultured in medium 199 containing 15% FBS, antibiotics, L-glutamine, sodium pyruvate, and EC growth factor as described previously [13]. HUVECs were used at passages 2 and 3 within 2 days after-confluence. 

### 2.4. Hemoglobin Preparation

Hb of different redox states, that is, (Fe^2+^) oxyHb, (Fe^3+^) metHb, and ferrylHb, was prepared as described [15]. Briefly, Hb was isolated from fresh blood drawn from healthy volunteers using ion-exchange chromatography on a DEAE Sepharose CL-6B column. MetHb was generated by incubation (30 min, 25°C) of purified Hb with a 1.5-fold molar excess of K_3_Fe(CN)_6_ over heme. FerrylHb was obtained by incubation (1 h, 37°C) of Hb with a 10 : 1 ratio of H_2_O_2_ to heme. After oxidation, both metHb and ferrylHb were dialyzed against saline (3 times for 3 hours at 4°C) and concentrated using Amicon Ultra centrifugal filter tubes (10,000 MWCO, Millipore Corp., Billerica, MA, USA). Aliquots were snap-frozen in liquid nitrogen and stored at −80°C until use. Purity of each Hb preparation was evaluated by SDS-PAGE followed by staining with ProteoSilver Plus Silver Staining Kit. The purity of Hb preparations was above 99.9%. Hb concentrations were calculated as described by Winterbourn [16].

### 2.5. Preparation and Oxidation of Low-Density Lipoprotein

LDL was isolated from the plasma of EDTA-anticoagulated venous blood of healthy volunteers by gradient ultracentrifugation (Beckman Coulter, Inc., Brea, CA, USA). The density of plasma was adjusted to 1.3 g/mL with KBr and a two-layer gradient was made in a Quick-Seal ultracentrifuge tube by layering saline on 10 mL plasma. Ultracentrifugation was performed at 302,000 g for 2 hours at 4°C (VTi 50.2 rotor). LDL samples were kept at −70°C until use and the protein concentration was determined by Pierce BCA protein assay Kit (Pierce Biotechnology, Rockford, IL, USA). Oxidation of LDL was carried out by the addition of heme (5 *μ*mol/L) and H_2_O_2_ (75 *μ*mol/L) as in our previous studies. In experiments assessing the LDL oxidizing potential of different Hb species EDTA-plasma was incubated for 1 hour at 37°C with different Hb species, that is, Hb, metHb, and ferrylHb, at a dose of 100 *μ*mol/L of Hb monomer prior to separation of LDL. 

### 2.6. Measurement of Lipid Peroxidation Products in LDL

The spontaneous oxidation of LDL treated as described previously was measured over a period of two-week incubation at 4°C by measuring the concentrations of conjugated dienes, lipid hydroperoxides, and thiobarbituric-acid reactive substances (TBARs). To assess conjugated diene content, LDL samples were diluted to 200 *μ*g protein/mL and optical density was measured at 234 nm. The method of Wolf was used to measure the total lipid hydroperoxide content in the LDL samples [14]. To measure TBARs, 50 *μ*L of a 200 *μ*g protein/mL LDL sample was mixed with 100 *μ*L of thiobarbituric acid reagent (0.375 g 2-thiobarbituric acid, 2.08 mL HCl, 15 mL 10% trichloroacetic acid to a final volume of 100 mL). After heating at 90°C for 20 minutes, the samples were cooled and extracted with 200 *μ*L n-butanol. The upper phase was measured spectrophotometrically at 532 nm. Results were calculated using a molar extinction coefficient of 1.56 × 10^5^ M^−1 ^cm^−1^ and are expressed as nmol TBARs/mg protein. 

### 2.7. Lipid-Mediated Oxidation of Hb

Purified Hb (20 *μ*mol/L heme) was incubated with H_2_O_2_ (200 *μ*mol/L), native or oxidized LDL (50–500 *μ*g protein/mL), or lipid derived from atheromatous lesion (LP) (500 *μ*g lipid/mL). Changes in Hb redox state were followed by UV-visible spectra recorded on a Beckman DU-800 spectrophotometer. 

### 2.8. EC Cytotoxicity Assay

Confluent HUVECs grown in 96-well tissue-culture plates were washed twice with PBS and exposed to heme and different Hb species, that is, Hb, metHb, or ferrylHb (10 *μ*mol/L heme) in HBSS. After 1 hour of incubation cells were washed and treated with H_2_O_2_ (75 *μ*mol/L) for 3 hours. Cell viability was assessed by MTT assay as described previously [17]. In separate experiments HUVECs were exposed to LDL (250 *μ*g protein/mL) which was treated with heme and different Hb species, that is, Hb, metHb, or ferrylHb (10 *μ*mol/L heme). Cell viability was measured by MTT assay after 6 hours of LDL exposure.

### 2.9. EC Monolayer Permeability Assay

HUVECs were cultured on gelatin-coated hanging cell culture inserts (Millipore Corporation, Billerica, MA, USA). After reaching confluence, cells were treated with heme, Hb, metHb, or ferrylHb (10 *μ*mol/L heme) for 12 hours. After treatment, fluorescein sodium salt (1 *μ*mol/L) in complete medium was added to the apical filter compartment, followed by a 60-minute incubation. Samples were collected from the lower basolateral compartment and fluorescence was measured by a fluorescence microplate reader at 488/525 nm (Synergy4, BioTek, Winooski, USA).

### 2.10. Monocyte Adhesion Assay

HUVECs were cultured on cover slips in 24-well plates and treated with heme, Hb, metHb, and ferrylHb (10 *μ*mol/L heme) for 12 hours. Peripheral blood mononuclear cells (PBMCs) were prepared by using Histopaque-1077. CD14 positive cells were then separated using a magnetic isolation procedure (MACS CD14 microbeads, Miltenyi Biotec GmbH, Bergisch Gladbach, Germany). Monocytes were suspended in serum-free DMEM to a concentration of 5 × 10^6^ cells/mL and incubated with Calcein AM (5 *μ*mol/L) for 30 minutes at 37°C. The labeled monocytes (10^5^ cells/well) were added to HUVECs in complete culture medium and incubated for 30 minutes at 37°C, followed by two washes with Ca^2+^- and Mg^2+^-containing HBSS to remove nonadherent cells. Cells were fixed with paraformaldehyde (3.7%, Merck) and blocked with donkey serum (5%, Jackson ImmunoResearch Europe Ltd., Suffolk, UK) for 30 minutes. After blocking, cells were stained with TRITC-conjugated phalloidin (25 *μ*g/mL) and with Hoechst (0.5 *μ*g/mL). Images were taken with a fluorescent microscope at a magnification of 400x (DM2500, Leica Microsystems GmbH, Wetzlar, Germany).

### 2.11. Quantitative Real-Time PCR (qRT-PCR)

Confluent HUVECs grown in 6-well plates were exposed to heme and different Hb species, that is, Hb, metHb, and ferrylHb (100 *μ*mol/L heme) in complete medium. After a 6-hour treatment cells were washed and total RNA was isolated using RNAzol STAT-60 according to the manufacturer's instructions (TEL-TEST Inc., Friendswood, TX, USA). Levels of heme oxygenase-1 (HO-1) and cyclophilin mRNA were measured by real-time RT-PCR. RNA was reverse transcribed by using Superscript II reverse-transcriptase (Invitrogen, Life Technologies Co., Carlsbad, CA, USA). Real-time PCR was carried out using the iCycler iQ Real-Time PCR System (Bio-Rad Laboratories, Hercules, CA, USA). The 25 *μ*L reaction mixture contained 0.3 nmol/L primers (for HO-1: + GGT-GAT-AGA-AGA-GGC-CAA-GAC-TG and − GGT-GTC-ATG-GGT-CAG-CAG-CT, for cyclophilin + ACG-GCG-AGC-CCT-TGG and − TTT-CTG-CTG-TCT-TTG-GGA-CCT), 0.13 nmol/L fluorescent probes (for HO-1 CTC-AAC-ATC-CAG-CTC-TTT-GAG-GAG-TTG-CAG, for cyclophilin CGC-GTC-TCC-TTT-GAG-CTG-TTT-GCA), 3 mmol/L MgCl_2_, 0.2 mmol/L dNTPs and 0.05 U/mL Taq DNA polymerase (Invitrogen, Life Technologies Corp., Carlsbad, CA, USA). Results are expressed as fold increase in HO-1/cyclophilin gene expression ratio compared to that of untreated cells.

### 2.12. Western Blot

HUVECs were cultured in 6-well plates and exposed to heme and different Hb species, that is, Hb, metHb, or ferrylHb (100 *μ*mol/L heme) in complete medium. After 8 hours of incubation the cells were solubilized in 10 mmol/L TrisHCl, containing 5 mmol/L EDTA, 150 mmol/L NaCl (pH 7.2), 1% Triton X-100, 0.5% Nonidet P-40, and protease inhibitors (Complete Mini, F. Hoffmann-La Roche Ltd., Basel, Switzerland). Proteins (20 *μ*g) were applied to 12.5% SDS-PAGE gels. After electrophoresis, proteins were transferred to a nitrocellulose membrane (Amersham Biosciences Corp., Piscataway, NJ, USA) and HO-1 was identified using a mouse anti-human HO-1 monoclonal antibody (Cat no. 374087, Calbiochem, Merck KGaA, Darmstadt, Germany) at a dilution of 1 : 2500. To ascertain equivalent protein loading, membranes were stripped and reprobed with a mouse anti-human GAPDH antibody (Cat no. N13300-221, Novus Biologicals, LLC, Littleton, CO, USA) at a dilution of 1 : 1000. To detect ferritins, samples were subjected to native gel electrophoresis. Anti-human ferritin H and L antibodies were a generous gift from Paolo Arosio. Primary antibodies were detected by using horseradish peroxidase-conjugated donkey anti-rabbit or goat anti-mouse IgG secondary antibodies. Antigen-antibody complex was detected by a horseradish peroxidase chemiluminescence system according to the manufacturer's instructions (Amersham Biosciences Corp., Piscataway, NJ, USA). Quantification was performed using video densitometry (AlphaDigiDoc RT, Alpha Innotech Corp., San Leandro, CA, USA). 

### 2.13. Detection of Covalently Cross-Linked Hb Multimers

Hb samples (1–4 *μ*g) were applied to 12.5% SDS-PAGE gels. After electrophoresis, proteins were transferred to a nitrocellulose membrane (Amersham Biosciences Corp., Piscataway, NJ, USA) and Hb was identified using a HRP-conjugated goat anti-human Hb polyclonal antibody (Cat no. ab19362-1, Abcam Plc., Cambridge, UK) at a dilution of 1 : 15000. In other cases cross-linked Hb multimers were detected by silver staining following SDS-PAGE. 

### 2.14. Statistical Analysis

Data are shown as mean ± S.D. Statistical analysis was performed by one-way ANOVA or Student's *t*-test as appropriate. *P* < 0.05 was considered significant.

## 3. Results

### 3.1. Oxidized Hemoglobin Species Induce Oxidative Modification of LDL

**Figure 1:**
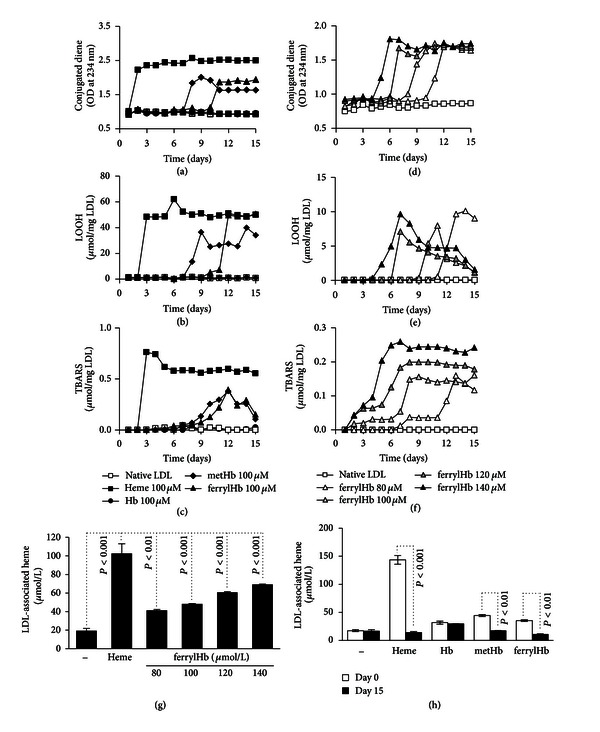
FerrylHb triggers oxidative modification of LDL. Human plasma was incubated with heme, Hb, metHb, and ferrylHb for 1 hour at 37°C, followed by separation of LDL. Concentrations are indicated and expressed as *μ*mol/L heme groups. Following separation samples were kept at 4°C. Conjugated dienes ((a) and (d)), LOOH ((b) and (e)), and TBARs ((c) and (f)) in LDL samples were measured every day for 15 days. Results are representative of 3 independent experiments. Heme contents of the same samples were measured on the day of separation (g) and 15 days later (h). Data represent mean ± S.D. of 3 independent experiments.

To model the possible interactions that could take place inside a complicated atherosclerotic lesion between lipids and different Hb species, we purified Hb from human blood and generated metHb and ferrylHb. We should note that ferrylHb is not a homogenous chemical entity but is a mixture of globin- and porphyrin-centered radicals (which can be very short-lived) and covalently cross-linked Hb multimers. Human EDTA-anticoagulated plasma was incubated with heme and the three different Hb species, that is, Hb, metHb, and ferrylHb (100 *μ*mol/L heme). After 1 hour of incubation at 37°C LDL was isolated by ultracentrifugation and oxidative modification of LDL was monitored by the formation of conjugated dienes, lipid hydroperoxides, and TBARs in samples incubated at 4°C for 15 days. Lipid peroxidation did not occur in LDL samples derived from nontreated or Hb-treated plasma samples (Figures 1(a)–1(c)). Starting on day 2 following-isolation, heme treatment caused extensive and rapid increase in conjugated dienes, LOOH, and TBARs content of LDL (Figures 1(a)–1(c)). MetHb, and ferrylHb also initiated oxidative modification of LDL and increased the levels of lipid peroxidation products at days 7–10 after isolation (Figures 1(a)–1(c)). The kinetics of formation of lipid peroxidation products in the LDL was strictly dependent on the dose of ferrylHb (Figures 1(d)–1(f)).

Heme released from oxidized Hb in plasma preferentially associates with LDL and is degraded shortly thereafter in the course of lipid peroxidation [14]. Therefore we assessed whether the heme moiety of ferrylHb is released and eventually taken up by LDL and degraded during lipid peroxidation. Plasma was incubated with heme or ferrylHb for 1 hour at 37°C followed by LDL separation and measurement of LDL-associated heme. As shown in Figure 1, ferrylHb treatment dose-dependently increased the concentration of LDL-associated heme in the LDL (Figure 1(g)). 

In order to determine whether similar events occurred in whole plasma, we treated fresh plasma with heme, Hb, metHb, or ferrylHb. Following the isolation of LDL, the concentration of LDL-associated heme was measured on the day of LDL isolation and 15 days later. We observed that LDL-associated heme underwent degradation when plasma was treated with heme, metHb, or ferrylHb (Figure 1(h)). In contrast the heme content of LDL derived from Hb-treated plasma did not change over a 15-day incubation period (Figure 1(h)). These results suggest that ferrylHb, like metHb, readily releases heme, following which iron is released upon oxidative scission of heme and serves to catalyze the process of lipid peroxidation. 

### 3.2. Oxidized Hb Species Trigger EC Death

**Figure 2:**
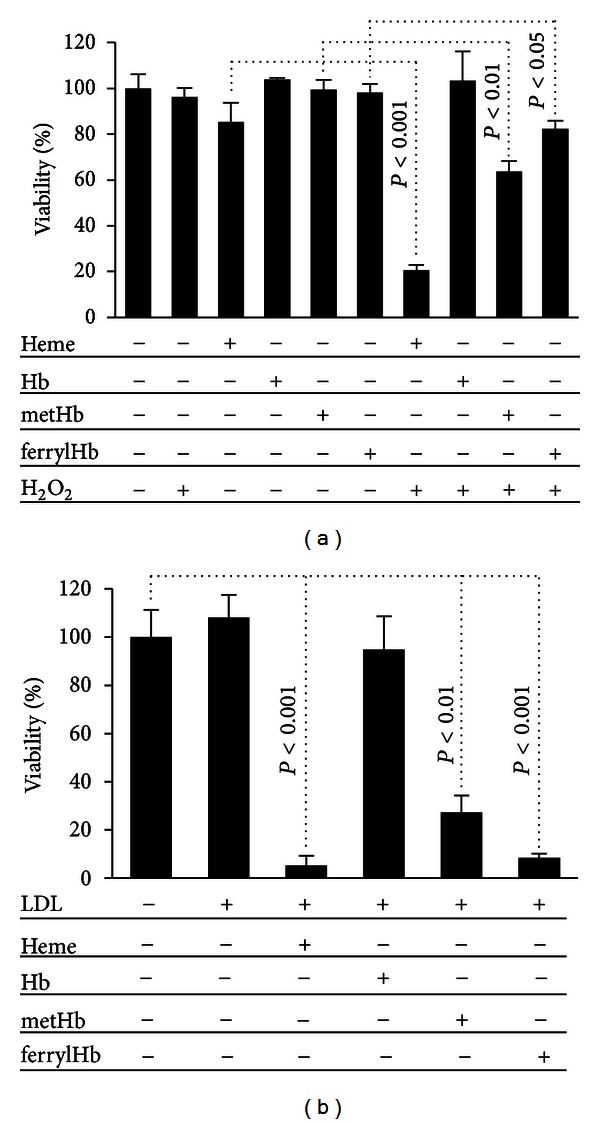
FerrylHb triggers EC death via different mechanisms. (a) Confluent HUVECs grown on 96-well plates were exposed to heme, Hb, metHb, and ferrylHb (5 *μ*mol/L heme) in HBSS for 1 hour. After washing with HBSS, cells were challenged with H_2_O_2_ (75 *μ*mol/L in HBSS) for 4 hours, followed by MTT assay to assess cell viability. (b) LDL was incubated with heme, Hb, metHb, and ferrylHb (10 *μ*mol/L heme) overnight. HUVECs were exposed to LDL samples at a dose of 250 *μ*g protein/mL for 4 hours. MTT assay was performed to determine cell viability. Results are shown as mean ± S.D. (*n* = 4) from one representative experiment of three.

We have shown previously that heme and metHb make EC more sensitive to oxidative stress by delivering redox active iron, and thus amplifying the generation of reactive oxygen species [13]. Therefore we tested whether ferrylHb, similar to metHb, can sensitize EC to oxidative stress. Confluent HUVECs were pretreated with heme, Hb, metHb, or ferrylHb at a dose of 5 *μ*mol/L heme. After 1 hour, heme-containing solutions were removed and cells were challenged with H_2_O_2_ (75 *μ*mol/L). Neither heme nor hemoglobins or H_2_O_2_ alone caused EC death. Moreover, no cytotoxicity was observed when Hb-treated cells were exposed to H_2_O_2_. In contrast, when HUVECs were pretreated with heme, metHb, or ferrylHb prior to H_2_O_2_ exposure, cell viability decreased (Figure 2(a)). This shows that while not cytotoxic *per se*, heme, metHb, and ferrylHb sensitize EC to H_2_O_2_-driven cytotoxicity. 

In our previous work we demonstrated that heme and metHb can exert cytotoxic effects on EC via oxidative modification of LDL [14]. To assess whether this is also the case with ferrylHb, LDL was incubated with heme, Hb, metHb, or ferrylHb (10 *μ*mol/L heme) overnight, and the resulting LDL was tested for cytotoxic effects. We observed that LDL samples treated with heme, metHb and ferrylHb became highly toxic to HUVECs (Figure 2(b)). In contrast, Hb did not generate cytotoxic LDL (Figure 2(b)). 

### 3.3. Oxidized Hemoglobin Species Induce HO-1 and Ferritin Expression in HUVECs

**Figure 3:**
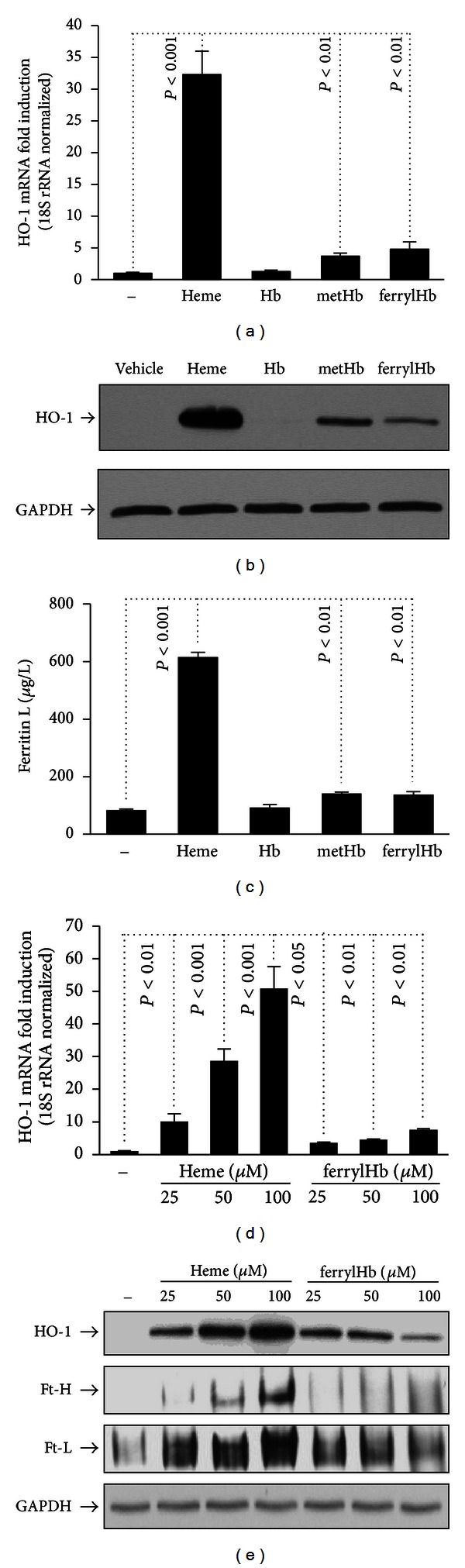
FerrylHb induces HO-1 and ferritin in EC. Confluent HUVECs grown on 6-well plates were exposed to heme, Hb, metHb, and ferrylHb (100 *μ*mol/L heme or as indicated in complete medium containing 15% of FBS). After 4 hours of incubation total RNA was isolated and HO-1 mRNA level was measured by quantitative RT-PCR (panels (a) and (d)). For protein expression, HUVECs were solubilized after 8 hours of treatment. HO-1 and ferritin H and L expression was detected by Western blot ((b) and (e)) or with ELISA (c). Immunoblots were reprobed with GAPDH and are representative of three independent experiments. Results are shown as mean ± S.D. (*n* = 3) from one representative experiment of three.

Upon exposure to free heme, EC upregulate the expression of HO-1 and H-ferritin to assure degradation of heme and safe storage of liberated iron, respectively. We have previously demonstrated that native (oxy) Hb does not induce HO-1 and ferritin in EC, whereas metHb does, because it releases its heme moiety [13]. We asked whether ferrylHb could transfer heme groups to the endothelium and thus upregulate HO-1 and ferritin synthesis. We observed that ferrylHb, similar to metHb, induces HO-1 mRNA and protein expression (Figures 3(a) and 3(b)). As with HO-1 expression, ferritin level was also increased in ferrylHb-treated cells compared to vehicle-treated controls (Figure 3(c)). These effects occur in a dose-dependent manner. However, when compared at a heme-molar ratio the effect of ferrylHb is lower than that of free heme (Figures 3(d) and 3(e)).

### 3.4. OxLDL and Reactive Lipid Mediators Derived from Complicated Atherosclerotic Lesions Initiate Hb Oxidation and Globin-Globin Crosslinking

**Figure 4:**
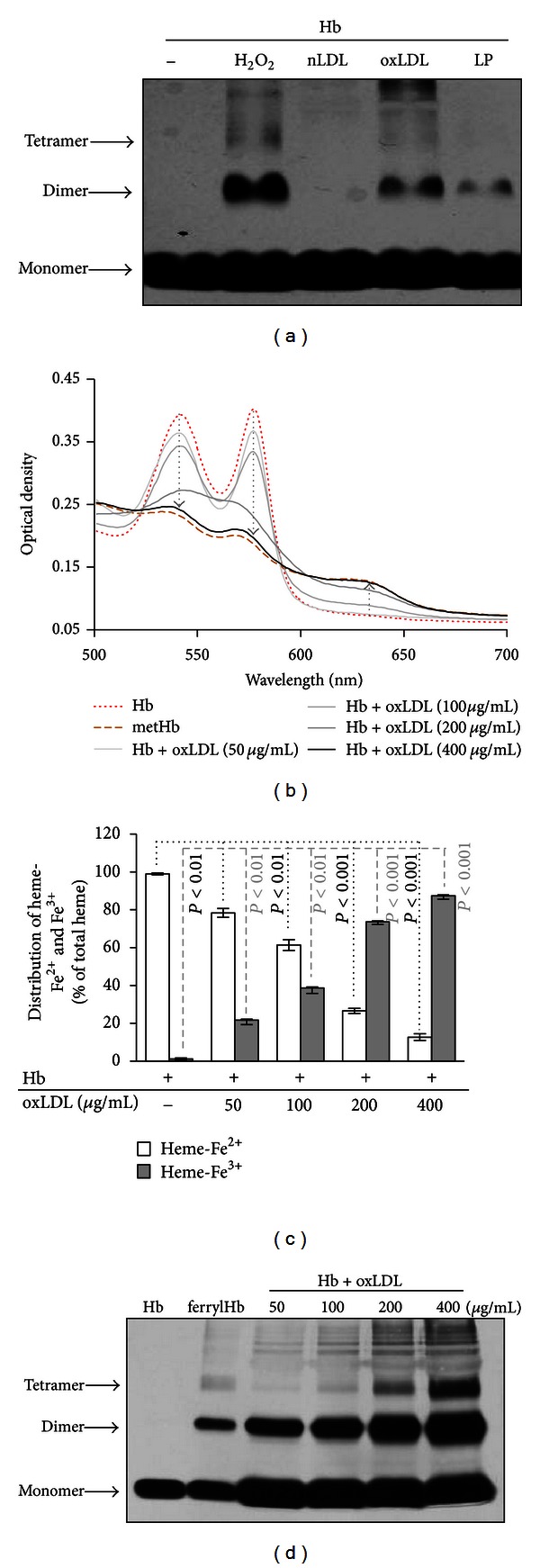
Oxidized LDL and atheroma lipids cause Hb oxidation and formation of ferrylHb. (a) Human Hb (20 *μ*mol/L heme) was treated with H_2_O_2_ (200 *μ*mol/L), native LDL (400 *μ*g/mL), oxLDL (400 *μ*g/mL), and lipid derived from human atherosclerostic plaque (LP) (400 *μ*g/mL). After 90 minutes of incubation Hb samples (4 *μ*g/lane) were subjected to SDS-PAGE followed by silver staining. ((b), (c), and (d)) Human Hb was incubated with oxLDL (50–400 *μ*g/mL) for 90 minutes. ((b) and (c)) Spectral scan of Hb samples were taken and concentrations of Hb and metHb were calculated based on the visible spectra. (d) Hb samples (4 *μ*g/lane) were subjected to SDS-PAGE followed by silver staining. Silver staining images and spectral scan are representatives of three independent experiments. Results are shown as mean ± S.D. (*n* = 3) from one representative experiment of three.

Lipid hydroperoxides, such as those found in oxLDL as well as in lipids derived from atheromatous lesions, can initiate Hb oxidation resulting in metHb formation and subsequent heme release [17]. Oxidized lipids and ferrylHb coexist in advanced atherosclerotic lesions but the role of reactive lipid mediators in the formation of ferrylHb and the subsequent crosslinking of Hb subunits has not been addressed. Therefore, we tested native and oxidized LDL, as well as lipids derived from human type IV atherosclerotic lesions, for their ability to induce Hb crosslinking, a surrogate marker of ferrylHb formation. Hb (20 *μ*mol/L heme) was treated with H_2_O_2_ (200 *μ*mol/L), native or oxidized LDL (500 *μ*g protein/mL), or lipids derived from human type IV atherosclerotic lesions (LP) (500 *μ*g lipid/mL). After a 2-hour incubation at 37°C, samples (4 *μ*g protein/lane) were subjected to SDS-PAGE followed by silver staining. Covalently cross-linked Hb dimer formation was observed in H_2_O_2_-treated as well as in oxLDL- and plaque lipid-treated Hb samples (Figure 4(a)). In contrast, native LDL did not induce Hb crosslinking (Figure 4(a)). Oxidation state of iron in the Hb modified by oxLDL was determined spectrophotometrically. OxLDL increased in a dose-dependent manner the percentage of Fe^3+^ Hb as assessed by an increased absorbance at *λ* = 630 nm accompanied by a decrease at *λ* = 577 nm and *λ* = 562 nm (Figures 4(b) and 4(c)). With increasing doses of oxLDL, dimer formation became more prevalent and at higher doses tetrameric and multimeric ferrylHb formation occurred as well (Figure 4(d)). 

### 3.5. Hb Oxidation and Crosslinking Induced by H_2_O_2_ and Reactive Lipid Mediators Can Be Inhibited by Haptoglobin (Hp) or GSH/GPx

**Figure 5:**
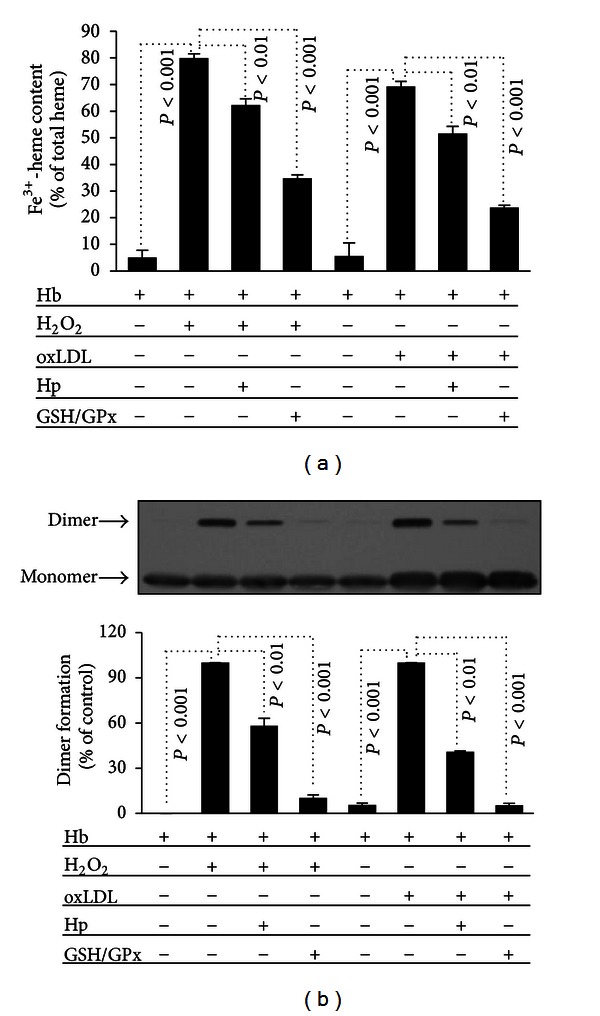
GSH/GPx and Hp inhibit H_2_O_2_ and oxLDL-mediated oxidation and crosslinking of Hb. ((a) and (b)) Human Hb (20 *μ*mol/L heme) was pretreated with Hp (50 *μ*mol/L) for 10 minutes at 37°C followed by 90 minute incubation with H_2_O_2_ (200 *μ*mol/L) and oxLDL (400 *μ*g/mL). Separately, H_2_O_2_ (200 *μ*mol/L) and oxLDL (400 *μ*g/mL) were pretreated with GSH/GPx for 10 minutes at 37°C followed by a 90-minutes of incubation with Hb (20 *μ*mol/L heme). (a) MetHb was determined spectrophotometrically. (b) Oxidation-induced dimer formation was monitored by Western blot. Immunoblot is a representative of three independent experiments. Results are shown as mean ± S.D. (*n* = 3) from one representative experiment of three.

Cell-free Hb binds to the acute-phase plasma protein Hp, promoting its endocytosis via the Hp receptor CD163, and thus preventing Hb accumulation in plasma [18, 19]. We tested whether binding of Hb to Hp inhibits ferrylHb formation. Hb oxidation was induced with H_2_O_2_ or oxLDL in the presence or absence of Hp. After 90 minutes Fe^3+^-heme content was determined spectrophotometrically (Figure 5(a)), and covalently cross-linked Hb formation was assessed by western blotting (Figure 5(b)). Hp slightly inhibited H_2_O_2_-mediated metHb formation. A more pronounced inhibitory effect of Hp was seen on Hb crosslinking; Hp inhibited by 42% and 60% covalently cross-linked Hb dimer formation in response to H_2_O_2_ and oxLDL, respectively. These results suggest that Hp has a role in suppressing the loss of Hb heme/iron and preventing the formation of covalently cross-linked Hb species. 

Lipid hydroperoxides in the oxLDL can trigger Hb oxidation, resulting in the formation of metHb [17], but the role of lipid hydroperoxides in Hb crosslinking has not been tested. To examine whether lipid hydroperoxides in oxLDL are responsible for oxLDL-mediated Hb crosslinking, we used glutathione-glutathione peroxidase (GSH/GPx) to decompose H_2_O_2_ and lipid hydroperoxides and assessed their effect on Hb crosslinking. GSH/GPx reduced the formation of metHb by 93% when Hb was oxidized by H_2_O_2_, and by 70% when Hb was exposed to oxLDL (Figure 5(a)). Exposing Hb to H_2_O_2_ or oxLDL in the presence of GSH/GPx led to 90–95% less dimer formation compared to Hb exposed to H_2_O_2_ or oxLDL in the absence of GSH/GPx (Figure 5(b)). These data highlight the critical role of lipid hydroperoxides in mediating Hb oxidation and subsequent covalent crosslinking of the Hb subunits. 

### 3.6. FerrylHb Increases EC Monolayer Permeability and Enhances Monocyte Adhesion

**Figure 6:**
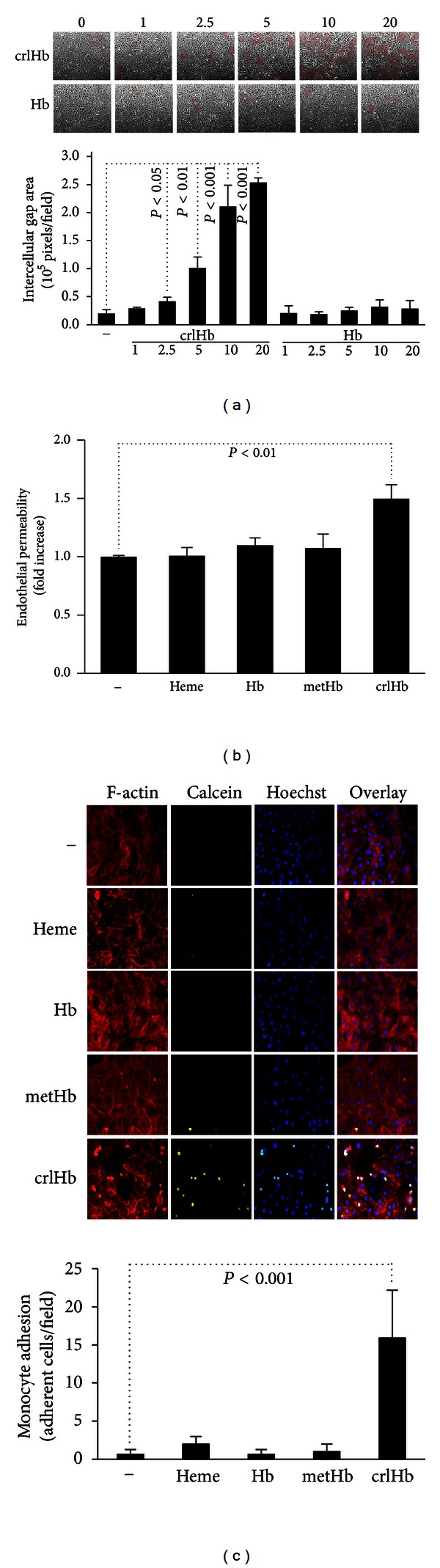
FerrylHb disrupts endothelial monolayer and induces leukocyte adhesion in HUVECs. (a) Confluent HUVECs grown in 6-well plates were exposed to Hb and ferrylHb at a dose of 0–20 *μ*g/mL overnight. Images are 100X, taken with an inverted microscope (Carl Zeiss 426126), and analyzed by ImageJ software. (b) Confluent HUVECs grown in hanging cell culture inserts were treated with heme, Hb, metHb, and ferrylHb (10 *μ*mol/L heme groups each) for 12 hours. Fluorescein (1 *μ*mol/L) was added into the apical filter compartment and was detected in the lower compartment after a 60-minute incubation. Endothelial permeability is expressed as fold increase over nontreated cells. (c) Confluent HUVECs were treated with heme, Hb, metHb, and ferrylHb (10 *μ*mol/L heme) for 12 hours. Monocytes were labeled and added to HUVECs (10^5^ cells/well) for 30 minutes at 37°C. Cells were stained with TRITC-conjugated phalloidin and with Hoechst. Images are 400x. Results are shown as mean ± S.D. (*n* = 3) from one representative experiment of three. Images are representative of 3 independent experiments.

We have previously demonstrated that ferrylHb activates EC *in vitro*, leading to the formation of intercellular gaps disrupting the endothelial monolayer [15]. Here we show that this effect is dose dependent (Figure 6(a)) and that intercellular gap formation is associated functionally with increased endothelial monolayer permeability (Figure 6(b)). This is a unique feature of ferrylHb as neither Hb nor metHb increased EC monolayer permeability. In our previous work we also showed that ferrylHb induces the expression of proinflammatory genes in EC [15]. Therefore we asked whether ferrylHb-triggered induction of these proteins—known to play a role in cell adhesion—is accompanied by increased monocyte adhesion to vascular EC. We found that human monocytes readily adhered to ferrylHb-treated EC. In contrast, treatment of EC with heme, Hb, or metHb did not promote monocyte adhesion (Figure 6(c)). These data suggest that the formation of ferrylHb may be a crucial event in the promotion of inflammatory responses. 

## 4. Discussion

The abundance of antioxidant enzymes and molecules makes the erythrocyte a relatively protective environment for Hb. Preventing or reversing Hb oxidation in RBCs is crucial. Under normal conditions, senescent RBCs are removed from the circulation by hemophagocytic macrophages of the reticuloendothelial system in a well-regulated way. Several pathological conditions are associated with intra- or extravascular release of Hb and subsequent increase of oxidative stress [20, 21].

 Hb oxidation leads to the formation of different Hb oxidation products, including ferryl iron containing heme, globin- and heme-centered radicals, covalently cross-linked heme-globin species and covalently cross-linked globin-globin multimers. In the literature these oxidized Hb species, are called, as a group, “ferrylHb.” These can be found in human blood under normal [22, 23] or pathological conditions [24]. We have previously reported the presence of covalently cross-linked globin-globin multimers of ferrylHb in human atherosclerotic lesions [8], but the involvement of ferrylHb in the pathogenesis of atherosclerosis remains to be further defined. 

Subendothelial retention of excess circulating LDL, oxidation of the trapped LDL, and immunological responses triggered by oxLDL are widely regarded as elements of atherogenesis (reviewed in [25]). Oxidation-specific epitopes are present on oxidized LDL particles, apoptotic cells and modified proteins in the vessel wall. It has been shown that elevated concentration of these epitopes predicts myocardial infarction, stroke and cardiovascular death [26]. Considering this close relationship between disease outcome and oxidative modifications it is highly important to characterize the potential oxidizing agents in the vessel wall. Hb can be a strong candidate that triggers oxidative damage in the atherosclerotic plaque. 

The two mechanisms via which Hb can enter to the atherosclerotic plaque area are (i) plaque rupture or (ii) intraplaque hemorrhage; the latter is common in advanced coronary atherosclerotic lesions [27–29]. Intraplaque hemorrhage is most likely originated from immature neovessels invading the atherosclerotic plaques as a result of its angiogenic activity [30, 31]. Recently, intraplaque hemorrhage has been linked to plaque vulnerability and considered as a critical event in triggering atherosclerosis-associated acute clinical symptoms [32]. 

Following intraplaque hemorrhage erythrocyte membrane proteins and iron accumulate in the plaque, suggesting that RBCs entering atherosclerotic plaques are lysed, their Hb is oxidized, and heme is released and degraded [4]. We have previously shown that both lipids extracted from human atheromatous plaques and oxidized LDL cause lysis of RBCs and subsequent Hb oxidation [8]. Theoretically these oxidized Hb species can initiate LDL modification via two distinct mechanisms with the involvement of (i) globin radicals or (ii) heme iron. For example, in a reaction system containing Hb, H_2_O_2_, and LDL, lipid peroxidation is rapid leading to the formation of conjugated dienes in less than 1 hour in the presence of 200 *μ*g/mL LDL, 3 mmol/L heme, and 4.5 mmol/L H_2_O_2_ [33]. This reaction has been shown to be triggered by globin radicals produced in the reaction of Hb and H_2_O_2_ [33]. On the other hand, heme release from oxidized Hb can also contribute to LDL oxidation. This notion is supported by the facts that (i) heme itself is a potent inducer of LDL oxidation, (ii) metHb, that does not have radical properties but releases heme moieties and initiates LDL oxidation and (iii) heme and Hb-initiated oxidation of LDL is inhibited by the heme-binding protein, hemopexin [34, 35]. In parallel with previously reported findings [33] here we demonstrated that ferrylHb can trigger LDL oxidation. We showed that heme derived from ferrylHb has been associated with LDL and caused a slow lipid peroxidation in 12 days in the presence of 1 mg/mL LDL and *≈*50 *μ*mol/L heme (Figure 1). During this period of time LDL-associated ferrylHb-derived heme underwent degradation similar to that of heme which is degraded during heme-mediated LDL oxidation. These results suggest that ferrylHb, depending on the circumstances of its production and the environment, can initiate LDL oxidation via two distinct mechanisms in which globin radicals and heme play the major roles. 

Hydrogen peroxide is the most studied reactive oxygen metabolite that can induce oxidation of Hb in cell-free systems [36, 37], as well as in intact erythrocytes [38]. In these complex reactions different Hb species are formed, including metHb and ferrylHb [9, 10]. Ferryl iron is unstable and reacts with specific amino acids of the surrounding globin chains [11, 12] resulting in the formation of globin radicals. Subsequent reactions between globin radicals yield covalently cross-linked Hb multimers [39]. Besides classical reactive oxygen metabolites, organic peroxides were shown to induce the leakage of Hb from human erythrocytes [40] and the generation of metHb [41]. FerrylHb generation was detected in the interaction between ruptured erythrocytes and LDL [42, 43]. Parallel with these findings here we showed that oxLDL but not native LDL caused Hb oxidation and subsequent covalent crosslinking of Hb subunits (Figure 4.) We found that formation of crosslinked Hb species is dose dependent, in a way that higher doses of oxLDL result in the formation of larger multimers with higher molecular weights. We found that pretreatment of oxLDL with GSH/GPx that converts lipid hydroperoxide to alcohol inhibited Hb oxidation and concomitant crosslinking suggesting the involvement of lipid hydroperoxides in the generation of these species (Figure 5).

In case of intravascular or extravascular hemolysis or hemorrhage the deleterious effects of cell-free Hb are thought to be controlled mainly via the action of Hp [44], that binds to Hb with high affinity [18, 45] and promotes its clearance by monocytes and resident macrophages of the reticuloendothelial system via scavenger receptor CD163 [19]. CD163-mediated endocytosis of Hp-Hb complex is followed by rapid induction of HO-1 that degrades heme into CO, biliverdin, and iron [21]. Concomitant with HO-1 induction, ferritin is upregulated to store the iron released from heme [19, 46]. Colocalized expression of CD163 and HO-1 was reported in a subpopulation of macrophages in neovascularized atherosclerotic lesions [46], as well as in lesions with intraplaque hemorrhage [47]. This macrophage subpopulation degrades Hb more quickly and produces less reactive oxygen species and more of the anti-inflammatory cytokine IL-10. Based on these features this hemorrhage-associated macrophage subpopulation was suggested to act in an anti-inflammatory and atheroprotective manner [46, 48].

Recently it has been shown that oxidative crosslinking of Hb is associated with reduced Hp binding; therefore, the endogenous Hp-CD163 scavenger pathway is impaired [39]. As a result of inadequate uptake of these structurally altered Hb species, macrophages fail to induce HO-1, a stress responsive enzyme that provides the anti-inflammatory and atheroprotective effects of hemorrhage-associated macrophages [39, 48]. Also, impaired uptake of ferrylHb might result in the release of free heme which induces programmed necrosis or apoptosis of macrophages [49] as well as other cells [50]. 

Besides facilitating the removal of cell-free Hb from circulation, Hp has been shown to prevent Hb oxidation as well as heme loss from oxidized Hb [33, 51, 52]. Cooper et al. explored the mechanism underlying the protective effect of Hp in peroxide-mediated Hb oxidation. Interestingly, they found that in the presence of Hp the steady-state concentration of ferryl iron increases, while lipid peroxidation is inhibited. This is because Hp binding stabilizes the ferryl iron as well as the globin radical located on tyrosine 145 [53]. In agreement with these findings we reported here that oxLDL-mediated Hb oxidation and formation of covalently crosslinked Hb multimers are inhibited by haptoglobin (Figure 5). 

EC activation and damage are associated with the initiation and progression of atherosclerosis [54]. Oxidation of Hb can harm endothelial cells in different ways. Heme released upon Hb oxidation can sensitize EC to oxidant-mediated killing [13] and can induce EC death via triggering oxidative modification of LDL [14, 55]. Here we demonstrated that ferrylHb exerts cytotoxic effect towards the vascular endothelium, mainly, via inducing oxidative modification of LDL, as ferrylHb slightly sensitizes EC to H_2_O_2_-mediated killing. Heme release is crucial in cellular responses triggered by metHb. When challenged with metHb, EC upregulate HO-1 to catabolize heme and ferritin to store liberated iron in a redox inactive form. We found that ferrylHb, similar to metHb, induces HO-1 and ferritin expression in EC. This suggests that heme release is not impaired by covalent crosslinking of globin chains in ferrylHb. Recently we showed that Hb oxidation via the generation of ferrylHb exerted proinflammatory effects on vascular EC [15]. In response to ferrylHb, EC rearrange their actin cytoskeleton leading to intercellular gap formation [15]. FerrylHb induces the expression of proinflammatory genes, for example, *E-selectin, Icam-1,* and *Vcam-1* in EC [15]. Here we demonstrated that gap formation is associated with increased endothelial permeability, and that elevated expression of adhesion molecules led to increased number of adherent monocytes on the surface of EC (Figure 6). Free heme and metHb do not activate EC, suggesting that heme release does not play a role in the ferrylHb-mediated inflammatory response. Furthermore Hb is also unable to induce EC, suggesting that the cross-linked species of ferrylHb act as an important and unique proinflammatory agonist.

## 5. Conclusion

In conclusion, we demonstrated that ferrylHb containing covalently cross-liked Hb multimers can be formed in atherosclerotic lesions by the interactions of Hb and reactive lipid components, mainly lipid hydroperoxides, in the plaque. There are similarities and differences between metHb and ferrylHb. Both Hb oxidation products can release heme, sensitizing EC to oxidant-mediated killing and initiating lipid peroxidation of LDL. On the other hand, FerrylHb is unique in that it acts as a proinflammatory agonist by targeting vascular EC. This activation results in increased EC monolayer permeability and enhanced monocyte adhesion. Taken together, interactions between cell-free Hb and atheroma lipids provoke a vicious cycle promoting the oxidation of plaque lipids and Hb which in turn trigger endothelial activation and cytotoxicity.

## Abbreviations

Hb: Hemoglobin
metHb: Methemoglobin
ferrylHb: Ferryl hemoglobin
LDL: Low-density lipoprotein
oxLDL: Oxidized LDL
EC: Endothelial cell
GSH: Glutathione
GPx: Glutathione peroxidase
RBC: Red blood cell
HUVEC: Human umbilical vein endothelial cell
LOOH: Lipid hydroperoxide
TBARs: Thiobarbituric acid reactive substances
HO-1: Heme oxygenase-1
Ft-H: Ferritin H chain
Ft-L: Ferritin L chain
GAPDH: Glyceraldehyde 3-phosphate dehydrogenase
HBSS: Hank's balanced salt solution
Hp: Haptoglobin
ICAM-1: Intercellular adhesion molecule-1
VCAM-1: Vascular cell adhesion molecule-1
LP: Lipid extracted from human atheroma.
